# Supplementary material for: Sintering Copper Nanoparticles with Photonic Additive for Printed Conductive Patterns by Intense Pulsed Light
Source: Nanomaterials (Basel). 2019 Jul 25;9(8):1071. doi: 10.3390/nano9081071 (PMC6723544; doi:10.3390/nano9081071)
Supplement: Supplementary file 1 [file nanomaterials-09-01071-s001.pdf]

Supporting Information for:

Sintering Copper nanoparticles with photonic additive for printed conductive patterns by intense pulsed light

Wan-Yu Chung[1], Yi-Chin Lai[1], Tetsu Yonezawa[2,], Ying-Chih Liao[1,*]*

[1] Department of Chemical Engineering, National Taiwan University, Taipei, Taiwan

[2] Division of Materials Science and Engineering, Hokkaido University, Sapporo, Hokkaido, Japan

Contents:

1. Thermogravimetric analysis of PVP.

2. UV-vis absorbance spectrum comparison of various composition.

3. SEM images of sintered Cu/CuO film with different sintering intensity.

Figure S1

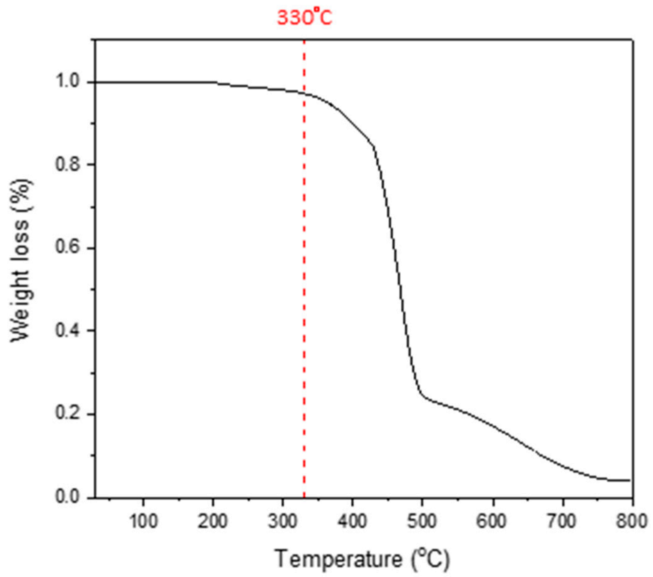

Figure S1 Thermogravimetric analysis of PVP.

Figure S2

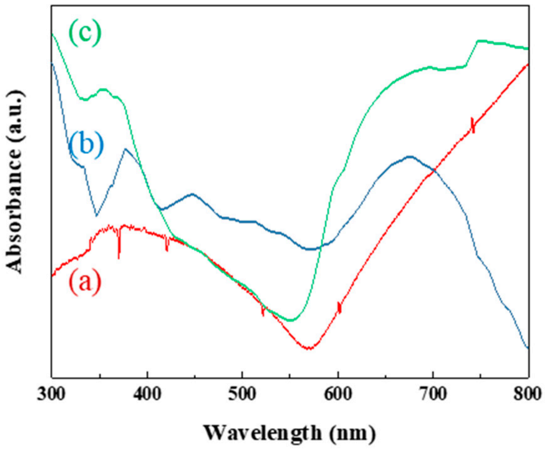

Figure S2. UV-vis absorbance spectrum comparison of (a) CuNP, (b)CuONPs and (c) CuONP/CuNP mixture with a weight ratio of 1/80.

Figure S3

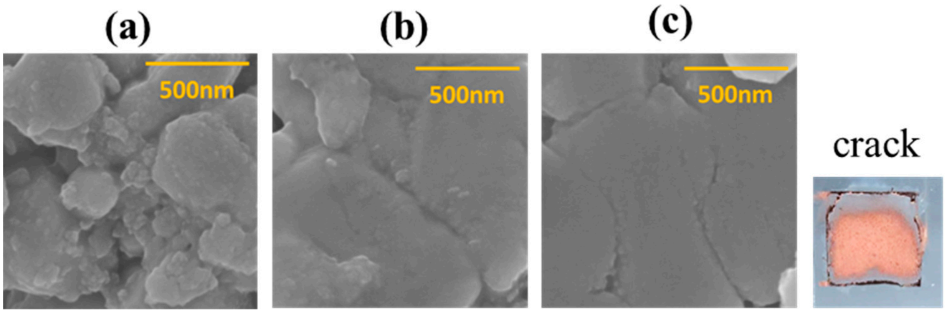

Figure S3. SEM images of sintered Cu/CuO film with sintering intensity at (a) 2.36J/cm^2, (b) 3.08 J/cm^2 and (c) 3.23 J/cm^2.
